# Can bone loss be reversed by antithyroid drug therapy in premenopausal women with Graves' disease?

**DOI:** 10.1186/1743-7075-7-72

**Published:** 2010-09-01

**Authors:** Tina Z Belsing, Charlotte Tofteng, Bente L Langdahl, Peder Charles, Ulla Feldt-Rasmussen

**Affiliations:** 1 Department of Endocrinology, National University Hospital, Rigshospitalet, Copenhagen, Denmark; 2 Department of Endocrinology, Aarhus University Hospital, Aarhus, Denmark

## Abstract

**Context:**

Hyperthyroidism can lead to reduced bone mineral density (BMD) and increased fracture risk particularly in postmenopausal women, but the mechanism behind is still unclear.

**Objective:**

Prospective examination of the influence of thyroid hormones and/or thyroid autoantibodies on BMD in premenopause.

**Design:**

We have examined 32 premenopausal women with untreated active Graves' disease from time of diagnosis, during 18 months of antithyroid drug therapy (ATD) and additionally 18 months after discontinuing ATD. Variables of thyroid metabolism, calcium homeostasis and body composition were measured every 3 months. BMD of lumbar spine and femoral neck were measured at baseline, 18 ± 3 and 36 ± 3 months. Data were compared to base line, a sex- and age matched control group and a group of patients with Hashimoto's thyroiditis treated with non-suppressive doses of levothyroxine.

**Results:**

The study showed significantly (p < 0.002) lower BMD in the thyrotoxic state compared to the control group with subsequent significant improvement during 18 ± 3 months of ATD compared to baseline (p < 0.001). However, during the following 18 months after stopping ATD femoral neck BMD decreased again unrelated to age (more than 0.4% per year, p < 0,002). The wellestablished effect of thyrotoxicosis on calcium homeostasis was confirmed. The positive predictor for best BMD was TSH receptor antibodies (TRAb) while free T4 correlated negatively in the thyrotoxic female Graves' patients (p < 0.02 and p < 0.003). In healthy controls and patients with treated Graves' disease both TSH and T4 correlated negatively to the bone mass (BMC) (p < 0.003).

**Conclusion:**

The results indicated a clinically relevant impact of thyroid function on bone modulation also in premenopausal women with Graves' disease, and further indicated the possibility for a direct action of TRAb on bones.

## Introduction

Thyroid hormones are known to stimulate bone turnover [1], and previous studies have shown decreased bone mineral density (BMD) [2] and increased risk of osteoporotic fractures [3-5] in patients with thyrotoxicosis [6]. However, many studies have been composed of a mixture of patients with different diagnoses (Graves' disease, toxic multinodular goiter, toxic solitary adenoma) and different patient characteristics (males, premenopausal females, postmenopausal females). Although all of these different thyroid diseases result in hyperthyroidism, as does also overtreatment with thyroid hormone replacement, the aetiologies and the course of the diseases are highly different. The extent and long-term reversibility of bone loss may therefore well depend on the aetiology of hyperthyroidism, but also on age and gender of the patients. A previous cross-sectional study of males and females with Graves' disease, found low BMD in women, but not in men [7], whereas a recent cross-sectional study also described low BMD in male patients with Graves' disease [8]. Menopause could theoretically aggravate the bone loss in thyrotoxicosis due to a synergistic effect of hypogonadism and excess thyroid hormone levels. However, the skeletal responsiveness to short-time stimulation with thyroid hormones in vivo has been demonstrated to be unaffected by menopausal status [9]. Furthermore, a cross-sectional study found no interaction between the effects of menopausal status and thyroid disease on BMD [10].

A few longitudinal studies have evaluated bone mass recovery after treatment of thyrotoxic women, demonstrating an only partly reversible bone loss [11-14]. In general, these previous studies were small, and one long-term study has shown a significant 11% increase in lumbar spine BMD three years after obtaining euthyroidism [15]. However, there is no complete concordance on the influence of thyrotoxicosis and antithyroid drug therapy on bone.

We chose to study a group of hyperthyroid patients with a single aetiology, namely Graves' disease, which is an autoimmune disease, and as one of the control groups we therefore chose another autoimmune thyroid disease, Hashimoto's thyroiditis, characterised by development of chronic hypofunction, which is then replaced with synthetic thyroid hormone. The aim of this prospective study was thus to evaluate long-term reversibility of BMD in premenopausal female patients with Graves' disease, during antithyroid drug therapy (ATD) and after discontinuation of the therapy, compared with an age-matched group of euthyroid female patients with Hashimoto's thyroiditis on stable and correct thyroxine replacement, and a female control group.

## Materials and methods

Thirty-two female premenopausal women newly diagnosed with Graves' disease and 18 euthyroid premenopausal patients with Hashimoto's thyroiditis treated with non-suppressive doses of levothyroxine (Eltroxin^®^) for a mean of 43 months (range: 0-360) were enrolled consecutively in the present prospective study. Nineteen healthy premenopausal women without thyroid disease or family history of thyroid disease served as age-matched controls. All were examined at the Department of Endocrinology, National University Hospital, Rigshospitalet, Denmark (Table 1).

**Table 1:** Demographic data of premenopausal female patients with Graves' disease compared to both healthy controls and patients with Hashimoto's thyroiditis on stable levothyroxine replacement.

	Controls	Graves' disease Months after diagnosis				Hashimoto's thyroiditis
**Study points (months)**		**Baseline**	**3**	**18 ± 3**	**36 ± 3**	
Number	19	32				18
Age (years)	43	39^NS^				43^NS^
	(37-49)	(34-45)				(36-50)
Height (m)	1.67	1.66^NS^				1.66^NS^
	(164-170)	(164-169)				(162-170)
Weight (kg)	67	59^#^	63*	65**	65**	67^NS^
	(62-73)	(55-63)	(59-67)	(61-70)	(61-70)	(63-71)
BMI (kg/m^2^)	24	21^#^	23*	23**	23**	24^NS^
	(22-26)	(20-23)	(21-24)	(22-25)	(22-25)	(22-26)
Pregnancies (no)	0	5				0
Abortions (no)	0	3				0
Smoking (no)	2	14				8
Birth control tablets (no)	0	1				1
Calcium tablets (no)	0	1				0
TAO (no)	0	5				0
Prednisone (no)	0	1				0

Data are expressed as antilog mean (CI 95%) and significance is marked as * = p < 0.05,

** = p < 0.005 when compared to baseline and marked differently as^# ^= p < 0.05, ^## ^= p < 0.005 when normal controls are compared to baseline data for patient with Graves' disease or patients with Hashimoto's disease. NS = non-significant. No=number of persons. TAO=thyroid associated ophthalmopathy.

Thyrotoxicosis was diagnosed by a high (above reference range) total thyroxine (T4) (reference range: 60-140 nmol/l), free thyroxine (fT4) (reference range: 9.1-23.8 pmol/l), total triiodothyronine (T3) (1.5-2.7 nmol/l) and/or free T3 (fT3) (reference range: 2.2-5.4 pmol/l) combined with low thyrotropin (TSH) (below detection limit) (reference range: 0.5-4.2 mU/l).

Five patients with Graves' disease had thyroid associated ophthalmopathy (TAO), one of whom was treated with a short course of prednisolone, 5 got pregnant during the study period (2 gave birth and 3 had an abortion), 14 were smokers and 1 was on birth-control pills. Eight patients with Hashimoto's thyroiditis were smokers and 1 on birth-control pills. In the control group there were 2 smokers and none was taking birth-control pills (Table 1). Neither patients nor controls had other concomitant diseases. The characteristics of the patients and controls are shown in Table 1.

The patients with Graves' disease were included in the study at diagnosis with baseline measurements before starting ATD, while all started treatment with beta-blocker (Propranolol^®^) for ethical reasons. The patients with Graves' diseases were otherwise treated solely with thiamazol (Thycapzol^® ^range: 2.5-20.0 mg per day). Patients with Hashimoto's thyroiditis continued their usual dose of levothyroxin (Eltroxin^® ^range: 50-200 µg, mean 1.45 μg/kg daily).

The patients with Graves' disease were examined every 3 months during an 18 months course of ATD followed by additional 18 months after stopping ATD (standard treatment regimen of the department). In case of relapse during the additional 18 months follow-up the patients were subsequently excused from the study. Patients with Hashimoto's thyroiditis and the normal healthy controls were investigated once.

All participants were seen in the outpatient clinic after an overnight of fasting. Serum levels of T4, fT4 T3, fT3, TSH, TSH receptor autoantibodies (TRAb), anti-thyroid peroxidase antibodies (anti-TPO), parathyroid hormone (PTH), total calcium (mmol/L) and Ca^2+ ^(mmol/L) were measured as well as urinary calcium excretion (mmol/24 hours) and body mass index (BMI, weight/height^2^). Body composition, as well as BMD and bone mineral content (BMC) of the femoral neck and lumbar spine were measured by Dual Energy X-ray Absorptiometry (DEXA). Serum and urine markers, BMI and body composition measurements were reported at 4 time points: baseline, 3 months, 18 ± 3 months and 36 ± 3 months. BMD and BMC of columnar spine and femoral neck were measured at 0, 18 ± 3 and 36 ± 3 months.

### Thyroid hormones and antibodies

Serum concentrations of TSH and peripheral thyroid hormones were measured by commercially available kits at the Clinical Biochemical Laboratory, National University Hospital, Rigshospitalet including serum concentrations of TSH measured by time resolved fluoroimmunoassay, (hTSH Ultra, Wallac, Turku, Finland), T4 by fluorescence polarization immunoassay (IM_X_^R^, Abbott Laboratories Diagnostics Div., Illinois, USA), and T3 by microparticle enzyme immunoassay (IM_X_^R^, Abbott).

The 2^nd ^generation DYNOtest TRAK^® ^(Brahms, Berlin, Germany) measures quantitatively antibodies against the human TSH receptor. The assay was calibrated according to WHO standard 90/672. Values below 1.0 IU/l were defined as negative, values above 1.5 IU/l positive and values between 1.0-1.5 IU/l considered questionably positive [16]. Samples from one subject were run in the same assay. The intra-assay coefficient of variation (CV) of 3 samples of 1.35, 6.2 and 29 IU/L were 15, 4 and 5%, respectively, and the inter-assay CV of 3 samples of 1.5, 21 and 29 IU/L were 15, 8 and 8%, respectively [17].

Anti-TPO was measured in duplicate by a luminescence immuno assay (LUMItest, BRAHMS Diagnostica, Berlin) at the Serum Institute, Department of Autoimmunity. All samples from one subject were run in the same assay. The range of the calibration curve was 60 to 3000 U/L [18].

### PTH and calcium assessments

PTH was measured in our laboratory as previously described [19,20]. Alkaline phosphatases (reference range: 80-275 U/L) were measured routinely by the Clinical Biochemical Laboratory, National University Hospital, Rigshospitalet as was total serum calcium (reference range: 2.2-2.6 mmol/L) and Ca^2+ ^(reference range: 1.15-1.35 mmol/L) using an ion-selective electrode (Konelab 30i, Thermo) and with a between run CV = 3%.

Patients and normal controls collected urine for 24 hours before each appointment. Calcium excretion (mmol/24 hour) was measured and creatinine clearance calculated (reference range: 0.8-2.5 ml/sec) by the Clinical Biochemical Laboratory, National University Hospital, Rigshospitalet.

### Lipid profile

Total cholesterol (reference range: 4.0-7.3 mmol/L), LDL cholesterol (0.9-1.9 mmol/L), HDL cholesterol (2.7-4.1 mmol/L) and triglycerides (0.5-2.20 mmol/L) were measured by the Clinical Biochemical Laboratory, National University Hospital, Rigshospitalet using an enzymatic colorimetric tests (MODULAR, P-modul, Roche) and with a between run CV = 5%.

### DEXA scans

DEXA measurements of body composition were performed as whole body scans using the Norland XR-26 MarkII/HS (Norland Corporation, WIS) with high-speed dynamic filtration as previously described [21]. Three compartments were measured separately: Total bone mass (BMC), total fat mass (TFM) and total lean body mass (TLM) the sum of which gives the total body weight. The accuracy was 0.2 kg for total soft tissue and 0.001 kg for the fat mass. During the study period the scanner was equilibrated daily against a phantom provided from the manufacturer [22,23]. BMD, corresponding to BMC (in g) related to the area of the bone and thus given in g/cm^2^, was calculated separately at the left femoral neck and lumbar spine (L2-L4). The femoral neck T-scores (number of SDs above or below young adult mean BMD) and Z-scores (number of SDs above or below the age-matched mean BMD) were based upon the reference data sets using Norland XR-26 analyses software provided by the factory [24,25]. The same laboratory technician performed all scans with an intra-operator variation of 5%. Height and weight were measured and BMI (kg/m^2^) was calculated.

### Statistics

Serum concentrations of thyroid hormones, thyroid antibodies, PTH, calcium, and body composition were not distributed normally, and therefore results were log_10 _transformed before parametric analyses. Z- and T-scores were not transformed. Differences between the normal control group and patients were measured using student's t-test. Differences from baseline (each patient being her own control) were tested using a one-way one factor repeated measures ANOVA and post hoc testing using both Newman-Keuls and/or Duncan's multiple range test. Multiple regression tests (forward stepwise) were performed to investigate possible predictors for BMD. Back transformed data are presented as mean and 95% confidence interval (CI 95%). By post hoc analyses 32 patients in each group were statistically sufficient to demonstrate a 10% difference in BMD between groups with a p-value of <0.02, and longitudinally within patients with a p-value of < 0.01. The analyses were performed using Statistica version 6.1, StatSoft Inc, USA.

#### Ethical considerations

All participants gave their written informed consent. The study was in accordance with the Helsinki declaration and all protocols were approved by the Danish Local Ethical Committee of Copenhagen, Denmark (approval no: KF-094/95, KF-247/98) as well as the Registration Committee (approval no: 1995-1200-133).

## Results

### Thyroid function and autoantibodies

Patients with Graves' diseases had had self-reported symptoms of thyrotoxicosis for a mean of 7 months (CI 95%, 5-10) before the time of diagnosis. At diagnosis the mean T4 level was 246 nmol/l (231-263), T3 was 5.4 nmol/l (4.6-6.3) and TSH was (< 0.01 mU/l (< 0.01-0.02)) (Table 2). Most patients with Graves' diseases obtained euthyroidism within 3 months. The patients were euthyroid after 18 ± 3 months of ATD with a mean T4 level of 98 nmol/l (91-105), T3 of 1.5 nmol/l (1.4-1.7) and TSH of 1.12 mU/l (0.67-1.88) (Table 2).

**Table 2:** Profile of the thyroid function and thyroid autoantibodies of premenopausal female patients with Graves' disease compared to both healthy controls and patients with Hashimoto's thyroiditis on stable levothyroxine replacement.

	Controls	Graves' disease Months after diagnosis				Hashimoto's thyroiditis
**Study points (months)**		**Baseline**	**3**	**18 ± 3**	**36 ± 3**	
Number	19	32				18
TSH (mU/L)	1.5	< 0.01^##^	0.2**	1.1**	0.6**	1.2^NS^
	(1.3-1.7)	(< 0,01-0.02)	(0.1-0.5)	(0.8-1.9)	(0.4-1.0)	(0.4-3.7)
T4 (nmol/L)	91	246^##^	93**	98**	103**	105 ^NS^
	(84-98)	(231-263)	(76-115)	(91-105)	(92-115)	(92-120)
fT4 (pmol/L)	14	51^##^	15**	14**	15**	16 ^NS^
	(14-15)	(45-56)	(13-18)	(13-15)	(14-16)	(14-18)
T3 (nmol/L)	1.5	5.4^##^	1.8**	1.5**	1.5**	1.6 ^NS^
	(1.3-1.7)	(4.6-6.3)	(1.5-2.2)	(1.4-1.7)	(1.3-1.7)	(1.5-1.8)
fT3 (pmol/L)	5.2	24^##^	5.8**	4.6**	4.2**	5.7 ^NS^
	(4.7-5.8)	(19-30)	(4.6-7.2)	(4.3-4.9)	(3.8-4.8)	(4.9-6.7)
TRAb (< 1.0	< 1.0	7.3^##^	4.1**	2.0**	1.2**	< 1.0
IU/L)		(4.9-10.8)	(2.6-6.2)	(1.3-2.9)	(0.8-1.8)	
Anti-TPO	< 60	292^##^	206^NS^	174*	249^NS^	1452^##^
(< 60 IU/L)		(166-513)	(121-349)	(107-282)	(143-435)	(785-2686)

Data are expressed as antilog mean (CI 95%) and significance is marked as * = p < 0.05, ** = p < 0.005 when compared to baseline and marked differently as ^# ^= p < 0.05, ^## ^= p < 0.005 when normal controls are compared to baseline data for patients with Graves' disease or patients with Hashimoto's disease. NS = non-significant.

TRAb and anti-TPO decreased significantly from 7 IU/L (5-11) at baseline to 1 IU/L (1-2) p < 0.00005 and from 292 IU/L (166-513) at baseline to 174 IU/L (107-282) p < 0.02, respectively. Patients with Hashimoto's thyroiditis had been treated with T4 for a mean of 43 months when entering the study and were euthyroid and with positive anti-TPO (1452 IU/L (785-2686) (Table 2).

### Body composition and lipid profile

The body weight of the patients with Graves' disease increased 6 kg (p < 0.00006 by conventional method) and 7 kg by DEXA measurement (p < 0.00005) 36 ± 3 months after start of ATD. The increased weight was mainly caused by an increase in muscle tissue, since TLM increased 4 kg (p < 0.00005), TFM only 3 kg (p < 0.003) and BMC 0.2 kg (p < 0.02) (data not shown).

The 3 fractions of cholesterol were lower and triglyceride higher when baseline values for the patient with Graves' disease were compared to the control group (data not shown). Patients with Hashimoto's thyroiditis had significantly higher triglyceride concentrations compared to the control group, but similar cholesterol levels. During the 36 ± 3 months study period the total, HDL and LDL cholesterol increased in patients with Graves' disease (p < 0.0001) while triglycerides decreased (p < 0.0004) (data not shown).

### Calcium assessments

In general, total serum calcium, Ca^2+ ^and calcium excretion were high at diagnosis of Graves' disease and decreased during 36 ± 3 months follow-up (Table 3). PTH increased significantly from 23 pg/ml (16-34) (mean (CI 95%)) to 39 pg/ml (25-59) at the end of ATD at 18 ± 3 months (p < 0.02). Serum Ca^2+ ^decreased significantly from 1.32 mmol/L (1.31-1.34) to 1.25 mmol/L (1.23-1.26) (p < 0.0001). Urinary calcium excretion decreased significantly from 5.2 (4.1-6.5) to 2.8 mmol/24 hours (2.2-3.7) (p < 0.0001) after 3 months and increased to 4.0 mmol/24 hours (3.1-5.2) after 36 ± 3 months follow-up. Alkaline phosphatases were all the time within the reference range (80-275 U/L) but in the upper end at diagnosis, increasing significantly to 251 U/L (218-288) during the first 3 months of ATD and fell again after 18 ± 3 and 36 ± 3 months (Table 3). The patients with Graves' disease had a normal creatinine clearance at all measuring points (Table 3). No significant differences were found in the patients with Hashimoto's thyroiditis (Table 3).

**Table 3:** Calcium assessments of premenopausal female patients with Graves' disease compared to both healthy controls and patients with Hashimoto's thyroiditis on stable levothyroxine replacement.

	Controls	Graves' disease Months after diagnosis				Hashimoto's thyroiditis
**Study points (months)**		**Baseline**	**3**	**18 ± 3**	**36 ± 3**	
Number	19	32				18
PTH^¤ ^(pg/ml)	39	23^NS^	32*	39*	-	32 ^NS^
	(22-72)	(16-34)	(22-46)	(25-59)		(13-83)
Calcium (mmol/L)	2.23	2.42^##^	2.30**	2.27**	2.31**	2.23 ^NS^
	(2.19-2.28)	(2.38-2.46)	(2.27-2.34)	(2.24-2.29)	(2.28-2.35)	(2.24-2.35)
Ca^2+ ^(mmol/L)	1.21	1.32^##^	1.23**	1.24**	1.25**	1.26 ^NS^
	(1.20-1.23)	(1.31-1.34)	(1.22-1.26)	(1.23-1.25)	(1.23-1.26)	(1.16-1.38)
Alkaline phosphatase	136	223^##^	251^##^	180**,^#^	160**	150^NS^
(U/I)	(120-155)	(199-249)	(218-288)	(159-204)	(141-180)	(126-179)
Urinary Calcium (mmol)	4.1	5.2^NS^	2.8**	3.6 ^NS^	4.0 ^NS^	3.4 ^NS^
	(2.9-5.9)	(4.1-6.5)	(2.2-3.7)	(2.9-4.5)	(3.1-5.2)	(2.6-4.5)
Creatinine Cl. (ml/sec)	1.3	1.2^NS^	1.3^NS^	1.2 ^NS^	1.3 ^NS^	1.1 ^NS^
	(1.2-1.5)	(1.1-1.4)	(1.2-1.4)	(1.1-1.4)	(1.1-1.5)	(1.0-2.3)

Data are expressed as antilog mean (CI 95%) and significance is marked as * = p < 0.05, ** = p < 0.005 when compared to baseline and marked differently as ^# ^= p < 0.05, ^## ^= p < 0.005 when normal controls were compared to baseline data for patient with Graves' diseases or patient with Hashimoto disease. NS = non-significant. ¤ = N varied from 4-11.

### Bone status

**Figure 1:**
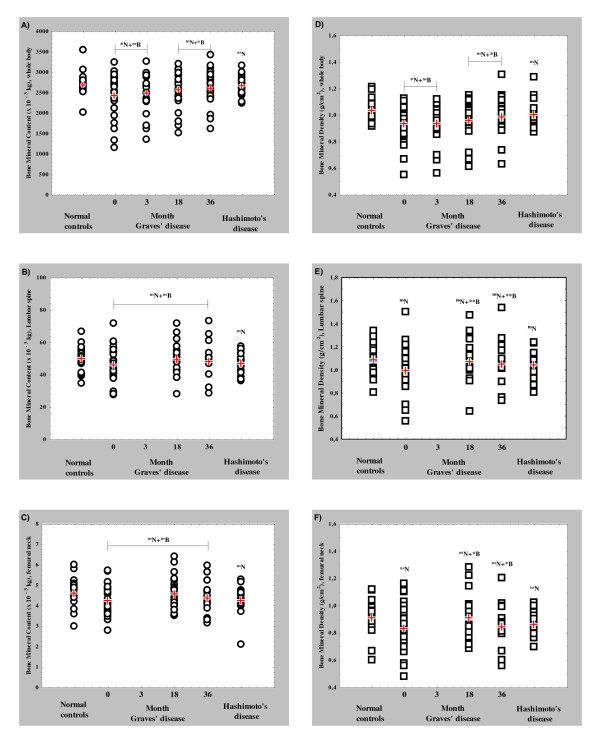
**Measurements of whole body (A), lumbar spine (B) and femoral neck (C) bone mineral content (BMC) (kg) as well as whole body (D), lumbar spine (E) and femoral neck (F) bone mineral density (BMD) (g/cm^2^) in female normal controls, patients with Hashimoto's thyroiditis and premenopausal women with Graves' diseases during 36 ± 3 months follow-up (at baseline, 3 and 18 ± 3 months on antithyroid drug therapy and additional 18 ± 3 months without antithyroid drug thyroid therapy or to relapse)**. + Represents back transformed mean. Significant differences to baseline (B) or to control group (N): * = p < 0.05 and ns = non significant.

The patients with Graves' disease had low whole body BMD (g/cm^2^), lumbar spine and femoral neck measurements at baseline compared to controls and all measurements increased significantly (p < 0.002) during the 18 ± 3 months of treatment with ATD (table 4, Fig 1). Femoral neck BMD (g/cm^2^), Z-scores and T-scores decreased significantly again (p < 0.001) after discontinuing ATD. Similar reductions were seen in BMC (Fig 1). The reduction was more than the expected age-related decrease (i.e. 0.4% per year) [25]. Few patients, however, fulfilled the criteria for osteopenia and/or osteoporosis. No significant differences were found between the patients with Hashimoto's thyroiditis, and the patients with Graves' disease, but there was a tendency towards low values in both groups compared to the control group (Table 4).

**Table 4:** Bone status (bone mineral density, BMD) of premenopausal female patients with Graves' disease compared to both healthy controls and patients with Hashimoto's thyroiditis on stable levothyroxine replacement.

	Controls	Graves' disease Months after diagnosis			Hashimoto's thyroiditis
**Study points (months)**		**Baseline**	**18 ± 3**	**36 ± 3**	
Number	19	32			18
whole body BMD					
Z-score	0.3	-0.2^#^	-0.1**	0.1**	0.2^NS^
	(-0.1 - 0.7)	(-0.5 - 0.1)	(-0.7 - 0.5)	(-0.5-0.6)	(-0.2 - 0.5)
T-score	0.1	-0.5^NS^	-0.1**	-0.3**	-0.1 ^NS^
	(-0.4 - 0.5)	((-0.9) - (-0.1))	(-0.7 - 0.5)	(-0.9 - 0.3)	(-0.4 - 0.3)
Lumbar spine BMD					
Z-score	0.6	-0.1 ^NS^	0.5**	0.6**	0.3 ^NS^
	(0.1-1.2)	(-0.6 - 0.6)	(-0.3 - 1.2)	(-0.4 - 1.6)	(-0.2 - 0.8)
T-score	0.1	-0.7 ^NS^	0.1**	-0.2**	-0.4 ^NS^
	(-0.5 - 0.8)	((-1.4) - (-0.1))	(-0.8 - 0.9)	(-1.3 - 0.9)	(-0.9 - 0.1)
Femoral neck BMD					
	0.91	0.83 ^NS^	0.91*	0.84*	0.86 ^NS^
	(0.84-0.98)	(0.77-0.90)	(0.8-1.0)	(0.75-0.94)	(0.82-0.90)
Z-score	0.5	-0.2^#^	0.3**	0.1**	0.1 ^NS^
	(0.04-0.95)	(-0.5 - 0.3)	(-0.3 - 0.9)	(-0.5 - 0.6)	(-0.4 - 0.5)
		-			
T-score	-0.1	-0.6 ^NS^	0.1**	-0.6*	-0.5 ^NS^
	(-0.6 - 0.5)	((-1.1) - (-0.1)) -	(-0.6 - 0.7)	(-1.3 - 0.2)	((-0.8) - (-0.2))

Z-scores and T-scores are expressed as (mean (CI 95%)) and significance is marked as * = p < 0.05, ** = <0.005 when compared to baseline and marked differently as ^# ^= p < 0.05, ^## ^= p < 0.005 when normal controls are compared to baseline data for patients with Graves' diseases or patients with Hashimoto's disease. NS = non-significant.

### Multiple regressions for BMD and BMC

Stepwise multiple regressions were performed to estimate the strongest predictor for whole body BMD and BMC. The model included age, BMI, Ca^2+^, alkaline phosphatase, cholesterol, HDL, LDL, triglyceride, TSH, total T4, fT4, total T3, fT3, TRAb and anti-TPO. In the normal control group the best negative predictor for BMD was TSH (β = -0.49, p < 0.03) and for BMC it was both TSH (β = -0.52, p < 0.03) and total T4 (β = -0.42, p < 0.03). This pattern was neither found in the patients with newly diagnosed Graves' disease nor in the patients with Hashimoto's thyroiditis. The best predictors for BMD in patients with Graves' disease were a negative association with fT4 (β = -0.55, p < 0.003) and a positive one with TRAb (β = 0.41, p < 0.02) while no significant predictors were found for BMC in this group (data not shown).

## Discussion

In this prospective study we have examined the bone metabolism in 32 premenopausal women with untreated active Graves' disease from time of diagnosis, during 18 months of ATD and to approximately 18 months after discontinuing their ATD. In order to avoid too many confounders and get a more uniform patient group than those previously studied we chose only females (males were excluded from this study due to their different body-composition compared to females), and only in premenopause (to avoid the postmenopausal bone loss), and only patients with the autoimmune Graves' disease. The obtained results and conclusions thus only apply to patients with these characteristics and cannot be extrapolated to all hyperthyroid patients. We measured thyroid and calcium parameters as well as BMC and BMD by DEXA scan with calculation of Z-scores and T-scores. Data were compared to baseline, an age matched control group and a group of patients with Hashimoto's thyroiditis (a different autoimmune thyroid disease) on non-suppressive doses of levothyroxine substitution in order to avoid suppressive effects from levothyroxine substitution on bones. The study showed significantly reduced whole body BMD in the thyrotoxic state compared to the control group, which improved significantly during 18 ± 3 months of ATD and 18 ± 3 months of post-ATD follow-up compared to baseline. However, 18 ± 3 months after stopping ATD femoral neck variables had decreased again probably due to a slightly higher level of thyroid hormones with a still decreased TSH as one possibility or an age related decline as another. However, the reduction was more than the estimated 2% age related decline as shown by Leslie et al [25] indicating an additional pathophysiological effect in the patients. In parallel, the well established negative calcium balance in hyperthyroidism [1] also improved with a decrease in serum calcium and urinary calcium excretion and an increase in PTH during ATD. At the time of thyrotoxicosis, we found that TRAb and fT4, reflecting the degree of autoimmune hyperthyroidism, were strong but opposite predictors of BMD.

The first case of thyrotoxic osteopathy was reported in 1891 by von Recklinghausen [26]. Since then numerous studies have addressed this issue, but for a variety of reasons many of them were not able to clarify the effects of thyroid hormones on skeletal integrity [27]. In general, the published clinical studies used different study designs such as a variable inclusion of men and pre- and postmenopausal women; the case-control studies have not been consistently and appropriately matched; both thyroid replacement and suppression therapies have gradually been changed over the last 20 years making results from present studies difficult to compare with historical data; different methods for assessment of BMD have been used and measurement of various skeletal sites using different techniques often produced incomparable results; finally, in one of the studies patients with heterogeneous thyroid diseases were investigated [27].

Recently, a large population based case-control study of 124,655 persons found an increased fracture risk in a subgroup of thyroid patients within 5 years from the diagnosis of hyperthyroidism and within 10 years from the diagnosis of hypothyroidism, thus supporting the notion that thyroid function is important for bone health [28]. However, several relevant confounders, known to affect bone mineral content and density, such as body weight and smoking were not taken into account.

In the present study all patients with Graves' disease were rather homogeneous. All were premenopausal women diagnosed at the first onset of Graves' thyrotoxicosis, and followed prospectively up to 36 ± 3 months after the diagnosis, and all were given a titration dose of thiamazole. They were furthermore compared to an age, gender and BMI matched control group. However, despite careful monitoring of the patients to maintain euthyroidism on ATD it cannot be excluded that the patients had shorter periods of thyroid dysfunction during or after the treatment with ATD, although this is less likely to occur in patients with Graves' disease than in multinodular goitres or toxic adenomas. It is, however, not a probable explanation for the reduction in BMD at the femoral neck between stopping ATD at 18 ± 3 and 36 ± 3 months. In addition, it cannot be excluded that confounders such as pregnancies, abortions, prednisolone, birth-control pills, other estrogens and smoking may have influenced the result. Overall, however, BMD was significantly decreased at the time of diagnosis and increased during ATD which is in keeping with previous studies [27,28].

The mechanisms for the decreased BMD in patients with hyperthyroidism have been shown to include an increased bone turnover, leading to a negative bone balance, with consequent expansion of the remodelling space, a decreased cortical thickness [28-30], and increased risk of trabecular perforations [1] The effects of thyroid hormones on bone turnover are presumably at least in part mediated by thyroid hormone receptors (TR) [31] although the molecular mechanisms are still unclear (30). TSH may influence bone turnover directly, since both high levels of endogenous TSH [28,32-34] and injection of recombinant TSH (Thyrogen^®^) [35] have been demonstrated to inhibit bone resorption and TSH receptors have been found on human osteoblasts [36]. TSH might also stimulate the expression of type 2 iodothyronine deiodinase in osteoblasts [37]. Thus, suppressed TSH levels as seen in hyperthyroidism might contribute to bone loss. However, Bassett and his group recently demonstrated in mice that bone loss in thyrotoxicosis was independent of circulating TSH levels and predominantly mediated by TRα [38]. Thus, additional studies are required to confirm the diffential role of TSH and/or T3 in thyrotoxic bone turnover.

In the present study TSH and T4 levels were both negatively associated with BMD in healthy controls, possibly reflecting a physiological regulation of bone metabolism by TSH stimulating T4 production. Population studies have found conflicting results. Morris suggested a bone protective role from TSH in postmenopausal women [39], while Griemnes et al found no association between TSH and BMD [40].

Conversely, at the time of overt hyperthyroidism fT4 and TRAb displayed opposite relationships, supporting the notion that high thyroid hormone levels reduced BMD, while TRAb might protect from bone loss through its effect on the TSH receptor. These results contradicted those of Majima et al who studied 56 Japanese male patients with newly diagnosed hyperthyroid Graves' disease and found that both TRAb as well as T4 correlated negatively with a reduction in BMD [7,32,37]. This finding was, however, in keeping with Wakasugi et al [41] and Jodar et al [42] previously demonstrating a significantly negative correlation between TRAb and lumbar BMD in hyperthyroid patients, and Kumeda et al [43] reported that TRAb did not correlate with either fT3 or fT4, but correlated closely with bone metabolic markers in Graves' disease. Other studies have suggested that the past history of Graves' disease itself, and not the current thyroid function, was responsible for bone loss in women receiving long-term levothyroxine therapy [7,27].

There are thus several indications of a direct anabolic effect of TRAb on bone metabolism but it is yet not clear whether it reflects the hyperthyroid state or is present regardless of the thyroid function [7,41,43]. The other correlations on thyroid function effects on bones found in the present and some other studies are not readily explainable. Yet, it must be appreciated that hyperthyroidism not only affects thyroid hormone production, but also the metabolism of binding proteins and the activity of deiodinases [44]. Measurement of both total and free thyroid hormones will therefore be very method dependant, and in an unpredictable manner, which could invalid results and thus also correlations [45]. Finally, it cannot be excluded that the increase in muscle mass after attainment of euthyroidism can contribute mechanically to an increase in BMD/BMC [reviewed in [46]].

In summary, we found that bone mass and density were significantly reduced in premenopausal women with newly diagnosed Graves' disease with a marked improvement during ATD. At time of diagnosis serum calcium levels and calcium excretion were increased and PTH was decreased, as indicators of accelerated bone turn over, all of which normalised during ATD. The best predictors for BMD were opposite relationships to TRAb and fT4, respectively in the thyrotoxic female Graves' patients, and TSH alone in the control group. Although the mechanisms cannot be explained by the present results, they did indicate an important impact of both thyroid function and TRAb on bone modulation not only in postmenopausal, but also in premenopausal women. Attainment of euthyroidism partly reversed the adverse effect of hyperthyroidism on bones. This is a very important clinical finding, which will have an impact on the general management of these patients. However, longer follow-up studies in hyperthyroid patients of other aetiologies and more experimental studies in cells or animals will be required to clarify the mechanisms for bone reversibility in patients with thyroid dysfunction.

## Authors' contributions

TZB participated in the design of the study, carried out the patient management, performed the statistical analysis and drafted the manuscript. CT assisted in the statistical analyses, interpretation of results and finalising the manuscript. BLL participated in interpretation of results and writing of the discussion, PC participated in interpretation of results and writing of the discussion. UF-R participated in the design of the study, patient recruitment, interpretation of the results and drafting the manuscript. All authors read and approved the final manuscript.

## Declaration of Interest

The authors declare no conflict of interest that would prejudice impartiality of this scientific work
